# Network Optimization of CNT Yarn Sensor Based on NNIA Algorithm in Damage Monitoring of 3D Braided Composites

**DOI:** 10.3390/ma15238534

**Published:** 2022-11-30

**Authors:** Minrui Jia, Zhenkai Wan, Xiaoyuan Pei, Jianmin Guo, Weichen Bao, Liying Gong, Yan Liu, Jun Zhao

**Affiliations:** 1 Office of The Cyberspace Affairs, Tiangong University, Tianjin 300387, China; 2 Engineering Teaching Practice Training Center, Tiangong University, Tianjin 300387, China; 3 School of Textile Science and Engineering, Tiangong University, Tianjin 300387, China; 4 School of Mechanlcal Engineering, Tiangong University, Tianjin 300387, China; 5 Analytical & Testing Center, Tiangong University, Tianjin 300387, China; 6 Industrial Technology Research Institute, Tiangong University, Tianjin 300387, China

**Keywords:** CNT yarn sensor, optimal allocation of sensors, non-dominated neighborhood immune algorithm, 3D braided composite, damage monitoring

## Abstract

In order to solve the optimization problem of carbon nanotube (CNT) yarn sensor network embedded in three-dimensional (3D) braided composite materials and realize the structural health monitoring of internal damage of aerospace parts, the multi-objective optimization of the number and location of sensors was studied using non-dominated neighborhood immune algorithm (NNIA). Through the research of 3D six-direction braiding process, stress sensitivity of single CNT yarn sensor, and damage location of 3D braided composites, the number, position, and coverage constraint functions based on NNIA algorithm are constructed. In addition, the number and position of three-dimensional braided composite embedded CNT yarn sensors with different sizes are solved. Through the stress experiment and data analysis of damaged parts, it is proved that the optimized configuration result of CNT yarn sensor obtained by NNIA algorithm is suitable for the damage monitoring of 3D braided composites. The damage location error is less than 1 mm. This study lays a foundation for the establishment of damage source localization model of 3D braided composites.

## 1. Introduction

Three-dimensional (3D) braided composites have become one of the advanced load-bearing structural materials used in aerospace and other fields [1]. In the service process of composites, the damage is inevitable due to the impact of high pressure, high temperature, corrosion, and other environments. In particular, the delamination of the internal interface of the composite and the micro cracks in the matrix are not easily observed. This type of damage will continue to develop with time and become a major safety hazard of composite structures. Once an engineering accident occurs, it will bring huge economic damage and casualties to the society. Therefore, the monitoring of composite damage has become an urgent need of all sectors of society.

In the research on damage monitoring of 3D braided composite structures using structural health monitoring technology, the optimal configuration of sensors is the key research direction [2]. Traditional structural health monitoring technology mainly uses a built-in optical fiber sensor to collect various signals of 3D braided composites. However, this type of sensor has some disadvantages, such as brittleness of optical fiber, poor compatibility with composite materials, and large diameter [3,4]. However, CNT yarn inherits the excellent strength and modulus of carbon nanotubes and exhibits piezoresistive effect during tensile strain. It is an ideal embedded sensor with light weight, excellent mechanical properties, and high sensitivity [5,6]. It can be embedded in the interior of composites to form a sensor network for real-time and efficient monitoring [7]. Bai et al. embedded CNT yarn into three-dimensional (3D) braided composites with five-axis yarns, and carried out progressive damage accumulation experiments on composites. It is found that the resistance change in CNT yarn measured in situ has a good correlation with the dependent variable of composite materials, in order that CNT yarn can better monitor the progressive damage accumulation of 3D five-direction braided composites [8]. Pan et al. dispersed aqueous CNT ink into the yarns and developed a strain sensor that can monitor human subtle and violent activities in real time [9]. MengWang et al. embedded the carbon nanotube (CNT) buckypaper into composite materials to carry out monotonic and cyclic bending load experiments through studying it, in order to verify the feasibility of applying carbon nanotube (CNT) buckypaper to the health features of composites [10]. Gao et al. added CNT into glass fiber reinforced composites to establish monitoring sensor networks in epoxy resin matrix. The monitoring sensor network and acoustic emission were used to monitor the damage propagation process of glass fiber reinforced composites under impact load, and the functional relationship between resistance signal and damage area was deduced [11]. Li et al. calculated the length of CNT yarn embedded in three-dimensional six-direction braided composites with Bezier curve, and found, through experiments, that the error between the calculated length and the actual length was less than 1%, and when the tensile strain exceeded 2%, the resistance change in CNT yarn became non-linear [12]. However, with the increase in material size and the improvement in monitoring accuracy requirements, the number of CNT yarn sensors continues to increase, resulting in the number and weight of cables and supporting equipment connected to them. This brings technical challenges to the application of CNT yarn sensors for space vehicles that are very sensitive to weight [13].

The research on the optimal placement of sensors has developed from the empirical placement method to the optimal placement algorithm. Optimization algorithms are mainly divided into traditional optimization algorithms and non-traditional optimization algorithms [14]. Traditional optimization algorithms are mature in theory and widely used. Representative algorithms include: Identification error minimum criterion, controllability/observability criterion, model reduction criterion, modal strain energy criterion, etc. [15]. These algorithms need sufficient prior knowledge, are difficult to deal with the target noise, and have poor robustness. As a result, the error of the optimal configuration scheme is large and cannot meet the expected accuracy of the optimal configuration [16,17,18]. With the rise in artificial intelligence technology, intelligent optimization algorithm has become a good way to solve this problem. These algorithms include: Genetic algorithm (GA), swarm intelligence algorithm (ant colony, particle swarm), annealing algorithm, firefly algorithm, artificial immune algorithm, neural network algorithm, tabu search algorithm, and hybrid optimization algorithm [19,20,21,22,23,24,25]. The artificial immune algorithm proposed by de Castro et al. shows better effect in solving multi-objective optimization problems [26]. Moreover, Calos improved the multi-objective artificial immune algorithm to solve the problem of less constraints or weak non-linear constraints [27]. Jiao et al. proposed a dynamic immune genetic algorithm [28]. Furthermore, according to the phenomenon of multiple antibody symbiosis and a few antibody activations in the simulated immune response, non-dominated neighborhood immune algorithm (NNIA) is proposed. Due to its unique non-dominant neighbor selection method and proportional cloning, the algorithm enables individuals in sparse regions to have more opportunities to conduct heuristic search. It has the characteristics of high performance, easy convergence, and low complexity. In addition, it is a very effective multi-objective optimization algorithm [29].

In this paper, the optimal configuration model of the number and location of CNT yarn sensor networks embedded in 3D braided composites is studied. By studying the four-step three-dimensional six-direction braiding process, the stress sensitivity of CNT yarn sensor, and the stress concentration distribution law of the local damage source of three-dimensional braided composite materials, the NNIA algorithm model and the improved NNIA algorithm model for bubble sorting and screening of dominant antibodies are constructed. The optimization of the number and position of CNT yarn sensors is realized. On the premise of not reducing the monitoring accuracy, the number of sensors is as small as possible, which are embedded in the reasonable position of the woven material, reducing the overall weight of the structure and helping in its application in a wider range of fields.

## 2. Materials and Methods

### 2.1. Preparation of 3D Braided Preforms and Composites Embedded with CNT Yarn Sensors

**Figure 1:**
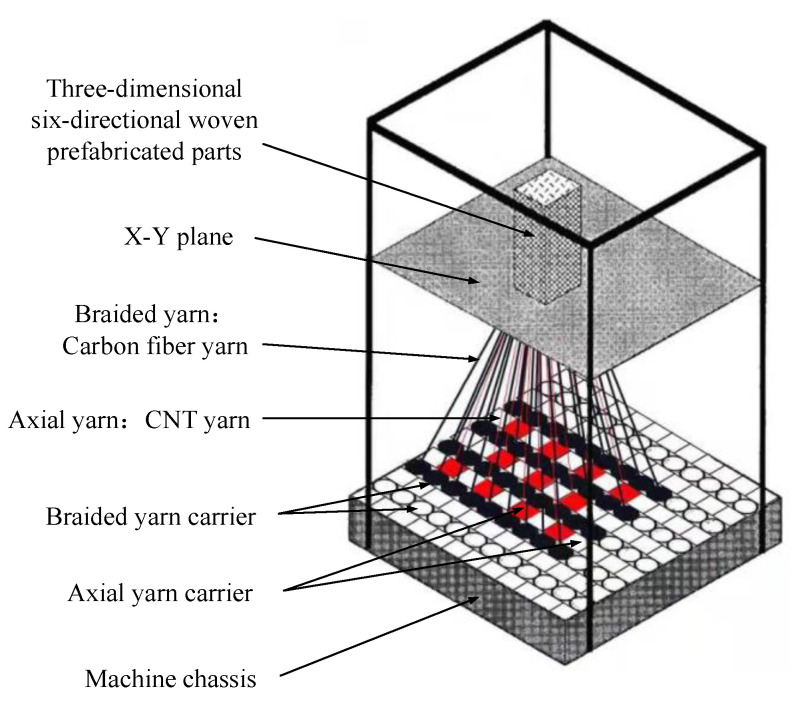
Schematic diagram of 3D braiding machine.

In the 3D four-direction braided composites, the bending of braided yarn will cause large errors to the monitoring results of CNT yarn sensors. A method of embedding CNT yarn sensor in 3D six-direction braided composites is proposed, which is consistent with the damage monitoring of composites. This realizes that the CNT yarn sensor is embedded in the three-dimensional braided composite material in a “linear” shape, and reduces the signal error caused by the bending of CNT yarn during the braiding process. The 3D six-direction braided composite was braided on the 3D braiding machine. The 3D braiding machine is shown in Figure 1.

**Figure 2:**
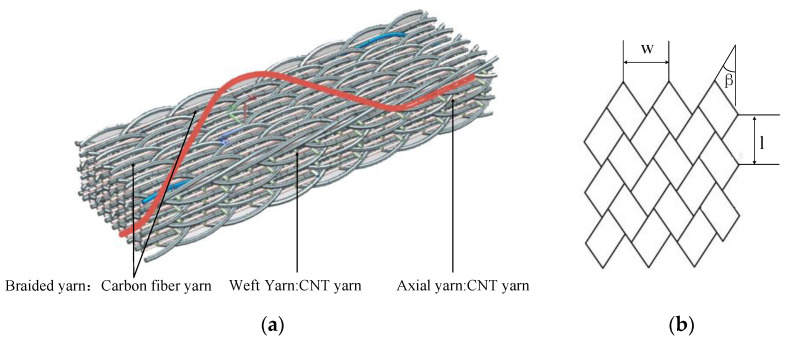
The 3D six-direction braided preform embedded with CNT yarn sensor: (**a**) The distribution of CNT in the preform; (**b**) the surface of the preform.

The yarn carriers are evenly distributed on the chassis of the 3D braiding machine, in which, ○ is a braiding yarn carrier, □ is a spindle yarn carrier. In the braiding process, one end of the braiding yarn and the shaft yarn (the fifth direction yarn) was first fixed on the hook above the chassis. According to the needs of the cross-sectional shape of the three-dimensional braided composite material, the other end is fixed on the hooks of the braided yarn carrier and the shaft yarn carrier of the chassis, respectively. On the chassis, the yarn carrier carries the yarn to move in a certain way. The yarn inter-braids with each other in the braiding direction to realize the whole braiding process. The 3D six-direction braided preform embedded with CNT yarn sensor prepared by the four-step method is shown in Figure 2. The distribution of CNT in the preform is shown in Figure 2a, and the surface braiding angle of the preform is shown in Figure 2b.

As can be seen in Figure 2a, during the braiding process, the red braided yarn was located inside the preform at one time and on the surface of the preform at another time. However, CNT yarn sensors, which are axial and weft yarn, always maintain a vertical or horizontal state throughout the braiding process. This feature enables CNT yarn sensors to have good positioning in 3D braided composites, providing favorable conditions for accurate detection. Along the braiding direction, an axial CNT yarn sensor was embedded in every other braided yarn; namely, the minimum distance that the axial sensor can be embedded is equal to the width (*w*) of the knot. A weft CNT yarn sensor can be embedded every 1/2 knot length (*l/2*) along the vertical braiding direction. The state of the width and length of the knots on the surface of the preform is shown in Figure 2b.

**Figure 3:**
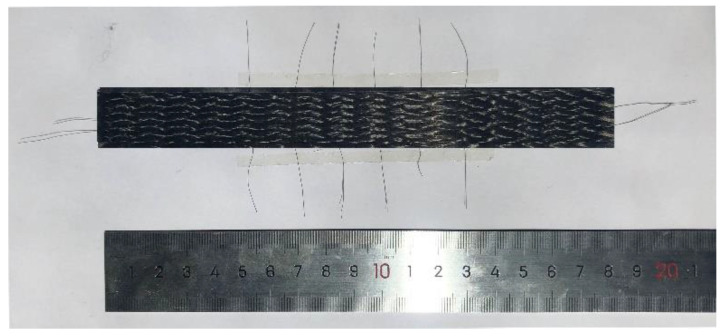
3D braided composites embedded with CNT yarn sensor.

The epoxy JL-155 and curing agent JH-196 were injected into the 3D braided composite preform embedded with CNT yarn sensor by vacuum assisted resin infusion to prepare the braided composites. The ratio and viscosity of the resin system are shown in Table 1. The specifications and parameters of specimen are shown in Table 2. The prepared 3D braided composites embedded with CNT yarn sensor are shown in Figure 3. The specification parameters of CNT are shown in Table 3. The solidify process of the specimen compared with Mr. Wu of our school is consistent, and the X-ray micro-computed tomography results adopted by Mr. Wu can specify the validity of specimen preparation [30].

### 2.2. Sensitivity Test of CNT Yarn Sensor

As a piezoresistive sensor, CNT yarn sensor is embedded in 3D braided composites, the stress sensitivity of which is one of the bases of NNIA algorithm modeling. CNT yarn sensors with diameter 100 μm were embedded in three-dimensional braided composites, and fracture tensile stress experiments were carried out. The regression fitting function is obtained by performing linear regression analysis on the stress resistance change rate data of *x* and, as shown in Formula (1).

The regression fitting function is obtained by performing linear regression analysis on the stress resistance change rate data of *x* and $e^{xt^{-1}}$, as shown in Formula (1):

(1) $$y=Ae^{xt^{-1}}+Bx$$

In Formula (1), x represents the stress; y represents the resistance change rate; A, B, and t are constants, in which, A=0.00344379, B=0.0000447032, t=238.1. Goodness of fit is $R^{2}=0.992414$. The function of the stress sensitivity of the CNT yarn sensor obtained by obtaining the first derivative on both sides of Formula (1) is:

(2) $$y^{\prime }=1.446\times 10^{-5}e^{0.0041992x}+4.47032\times 10^{-5}$$

When the value range of stress change *x* is [0 MPa, 600 MPa], the value range of resistance change rate $y^{\prime }$ is [5.92 × 10^−5^, 22.44 × 10^−5^]. With the increase in tensile stress, the stress sensitivity of the sensor increases exponentially. The value range of $y^{\prime }$ is the basis for determining the response boundary of the sensor.

### 2.3. Tensile Test of 3D Braided Composites Embedded with CNT Yarn Sensor

In order to fully verify the accuracy and effectiveness of the configuration scheme of CNT yarn sensor for damage location of braided composites, tensile stress experiments were carried out on 3D braided composites with no damage and typical damage embedded with CNT yarn sensor. The test refers to ASTM d3039 standard. The mechanical test equipment is SHIMADZU AG-250KN (SHIMADZU Company, Kyoto, Japan), and the tensile speed is 0.5 mm/min. The voltage amplifier composed of AD623ARZ chip is used to amplify the output signal of CNT yarn sensor 100 times, and then it is inputted into the structural health monitoring experimental system for analysis through A/D conversion. The size of all specimens are 200 mm × 200 mm × 4 mm.

**Figure 4:**
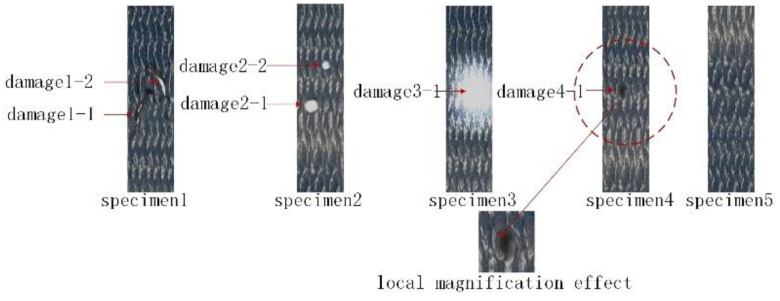
3D braided composite specimens with different damages and no damages embedded in CNT yarn sensor.

The 3D braided composite specimens with different damages embedded in the CNT yarn sensor are shown in Figure 4. Two damage points were set in specimen 1. Damage 1-1 was a one-dimensional linear damage with a length of 9.7 mm. The included angle with the horizontal position was 159°. The projection length in the axial direction was 9.07 mm, and the projection length in the latitudinal direction of the specimen was 3.48 mm. Damage 1-2 was a one-dimensional linear damage with a length of 14.1 mm. The included angle with the axial position was 41.7°, the projection length in the axial direction of the test piece was 12.1 mm, and the projection length in the latitudinal direction of the specimen was 7.3 mm. Two round hole damages were respectively set in test piece 2. Damage 2-1 was a round hole with a diameter of 3 mm and damage 2-2 was a round hole with a diameter of 5 mm. Specimen 3 was the impact damage caused by the Instron Dynatup9250HV falling weight impact test machine of Inst company. A hemispherical hammer with a diameter of 7.5 mm was used in the impact test. The falling weight was 6.5 kg. The maximum diameter range of damage was 14.6 mm. In addition, the damage numbers were named 3-1. The inside of specimen 4 had a small defect. The defect was a circle with a diameter of about 1.2 mm, which was enlarged in Figure 4. The damage numbers were named 4-1. As a comparative specimen, specimen 5 was not damaged. In order to ensure the repeatability of the test, 80% of the quasi-static failure load was taken according to the average value of the uniaxial tensile failure load, and 1024 cycles of tensile loading tests were carried out on these five specimens.

## 3. Stress Concentration Distribution Law and Location Principle of Damage Source in 3D Braided Composites

Under the action of external stress, there is a phenomenon of stress concentration at the damage location of 3D braided composites. This phenomenon is a prerequisite for the damage location of CNT yarn sensors. At the location of composite damage, the stress concentration is elliptical, and the distribution range is large along the axial direction and small along the weft direction. The mathematical model of stress concentration distribution obtained by curve fitting according to stress distribution data is shown in Formula (3):

(3) $$\begin{array}{c} P_{z}=\frac{P_{z_{\max}}(D_{z_{\max}}^{2}-9D_{z}^{2})\cdot e^{-\frac{3}{2}D_{z}^{2}(D_{z_{\max}}^{2}+\frac{600D_{z_{\max}}}{P_{z_{\max}}}-9D_{w}^{2})}}{D_{z_{\max}}^{2}}\\ P_{w}=\frac{P_{w_{\max}}(D_{w_{\max}}^{2}-D_{w}^{2})\cdot e^{-\frac{1}{6}D_{w}^{2}(D_{w_{\max}}^{2}+\frac{600D_{w_{\max}}}{P_{w}{}_{{}_{\max}}}-D_{w}^{2})}}{D_{w_{\max}}^{2}} \end{array}$$

In Formula (3), $P_{z}$ and $P_{w}$ respectively represent the stress value at a certain point when the stress concentration is distributed along the axial and weft directions. The vertical distance between the point and the center point of the damage source along the axial and weft directions of the specimen is defined as $D_{z}$ and $D_{w}$; $P_{z}{}_{{}_{\max}}$ and $P_{w}{}_{{}_{\max}}$ respectively represent the values of the stress concentration at the center point of the damage source along the axial and weft directions of the specimen; $D_{z}{}_{{}_{\max}}$ and $D_{w}{}_{{}_{\max}}$ respectively represent the maximum distance of stress concentration distribution along the axial and weft directions of the specimen.

Through the analysis of Formula (3), it is found that the value under stress concentration, i.e., $P_{z}{}_{{}_{\max}}$ and $P_{w}{}_{{}_{\max}}$, of the transverse and longitudinal CNT yarn sensors located on both sides of the damage source has the following relationship with the vertical distance from the source, such as $D_{z}$ and $D_{w}$, as shown in Formula (4):

(4) $$\frac{D_{1}^{2}}{D_{2}^{2}}=\frac{P_{1}}{P_{2}}\;in\;which,\;P_{1},\;P_{2}\leq P_{\max}$$

**Figure 5:**
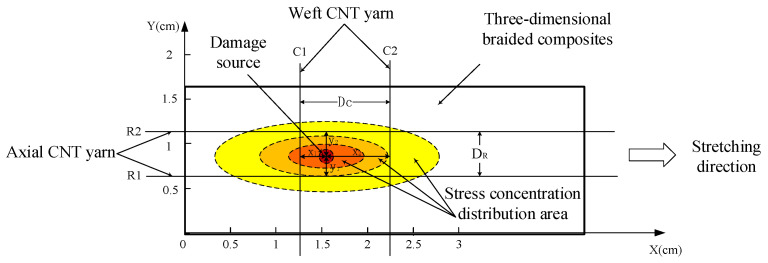
Schematic diagram of CNT yarn sensor damage location.

As an example, take the transverse sensors $C_{1}$ and $C_{2}$ as well as the longitudinal sensors $R_{1}$ and $R_{2}$, which are distributed around the damage source in Figure 5. In Formula (4), $P_{1}$ and $P_{2}$ represent the stress value of the stress concentration acting on the sensors on both sides of the damage source center; $D_{1}$ and $D_{2}$ represent the vertical distance between the two sensors and the damage source center. The subscripts 1 and 2 of $P_{1}$, $P_{2}$, $D_{1}$, and $D_{2}$, and the subscripts 1 and 2 of $C_{1}$, $C_{2}$, $R_{1}$, and $R_{2}$ all represent the sensor numbers on both sides of the damage source. The ratio of the vertical distance between the damage source and the sensors on both sides can be obtained by measuring the size of $P_{1}$ and $P_{2}$. If the vertical distance between the two sensors could be measured, the distance between the damage source and the two sensors can be calculated.

Based on the stress concentration distribution law of the damage source in Formula (4), the damage location principle shown in Figure 5 is adopted, and the coordinates of the damage source are assumed to be X and Y, respectively. The calculation Formula (5) of damage source coordinates is obtained through a series of calculations.

(5) $$\left\{ \begin{array}{l} X=X_{c_{1}}+\frac{D_{C}}{\sqrt{P_{w_{2}}/P_{w_{1}}}+1}\\ Y=Y_{R_{1}}+\frac{D_{R}}{\sqrt{P_{z_{2}}/P_{z_{1}}}+1} \end{array}\right.$$

In Formula (5), the induced stress concentration values are represented by $P_{W1}$, $P_{W2}$, $P_{Z1}$, and $P_{Z2}$, respectively; $X_{c_{1}}$ and $Y_{R_{1}}$ respectively represent the X and Y axis coordinate values of $C_{1}$ and $R_{1}$, respectively; $D_{R}$ and $D_{C}$ represent the distances between two adjacent sensors in the axial and weft directions, respectively, and these four quantities are known numbers.

## 4. Results and Discussion

### 4.1. Antigen Model Based on NNIA Algorithm for the Optimal Configuration of CNT Yarn Sensors

The principle of applying NNIA algorithm to solve the multi-objective optimal configuration problem of CNT yarn sensor number and position is to take the optimal configuration problem as an antigen and establish an objective function, and take the sensor number and position as an antibody and encode it. According to the sensor embedding process, sensitivity function, and damage location model, the constraint function was constructed. In addition, the optimal configuration scheme was obtained using NNIA algorithm. The objective function of the sensor network optimization problem was to find the minimum value of the total number of axial and weft CNT yarn sensors. The objective function is written as F(R,C), and the calculation formula is shown in Formula (6). In Formula (6), $f_{Z}(R)$ and $f_{W}(C)$ represent the total amount of sensors embedded in the axial and weft directions, respectively:

(6) $$\begin{array}{l} F(R,C)=\min f_{T}(R,C)\\ f_{T}(R,C)=f_{Z}(R)+f_{W}(C) \end{array}$$

### 4.2. Antibody Coding Based on NNIA Algorithm for Optimal Configuration of CNT Yarn Sensors

The NNIA algorithm takes the problem to be solved as an antigen, selects only a few relatively isolated non-dominant individuals as active antibodies, and performs proportional cloning according to their crowding degree to act on the antigen, and combines the constraints to screen the optimal antibody, which is the solution of the problem. The multi-objective optimization of the number and position of CNT yarn sensors as antigens is a problem to be solved. Antibody is an optimal configuration scheme for the number and location of sensors. Through the study of the process of embedding CNT yarn sensors into 3D six-direction braided composites, the number and position of sensors as antibodies were coded.

**Figure 6:**
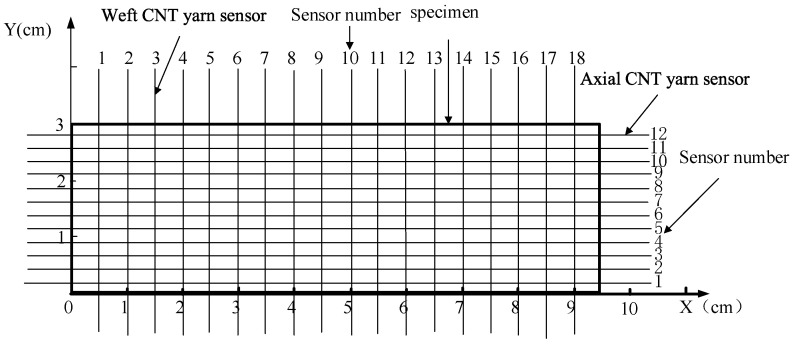
Numbering method of sensor embedded in braided composites.

The number of sensors is an important dimension of antibody. The number of CNT yarn sensors embedded along the axial and weft directions of the specimen can form a two-dimensional array. The elements in each cell in the array belonged to positive integers, such as {(2, 4), (4, 4), (8, 11)...}. The positions of all embeddable sensors can be coded from left to right and from bottom to top along the axial and weft directions of the specimen. The coding method is shown in Figure 6.

### 4.3. Constraint Function Modeling Based on NNIA Algorithm for Optimal Placement of CNT Yarn Sensors

#### 4.3.1. Constraint Function of CNT Yarn Sensor Number

Assuming the specimen size is L×W, according to the 3D six-direction braiding process, the constraint function of the number with the CNT yarn sensors embedded is shown in Formula (7):

(7) $$\left\{ \begin{array}{l} f_{Z}(R)\leq q-2=\frac{W}{w}-2\\ f_{W}(C)\leq p-2=\frac{L}{l}-2 \end{array}\right.$$

In Formula (7), p and q represent the maximum number of sensors that can be embedded along the weft and axial directions of the braided composites, respectively; h represents the knot length; and w represents the width of the knot. To ensure that the sensor was not exposed to the outside of the specimen after being embedded, the maximum number of sensors embedded in the axial and weft directions was subtracted from the number of sensors embedded at the edge of the specimen. The constraint function is one of the constraint functions for the number of sensors in the optimal configuration algorithm.

#### 4.3.2. Position Constraint Function of CNT Yarn Sensor

According to the damage location model of CNT yarn sensor, the embedded position of weft or axial sensors can be expressed by *x*-axis or *y*-axis coordinates, respectively; $\varpi (C_{i})$ and $\alpha (R_{j})$ are constraint functions of weft and axial sensor insertion positions respectively, as shown in Formula (8):

(8) $$\left\{ \begin{array}{l} \varpi (C_{i})=X_{C_{i}}\;in\;which,i\in [1,p-2]\\ \alpha (R_{j})=Y_{R_{j}}\;in\;which,j\in [1,q-2] \end{array}\right.$$

In Formula (8), $X_{C_{i}}$ and $Y_{R_{j}}$ are the coordinate values of the sensor in the weft and axial directions of the specimen, respectively; where i and j respectively represent the number of weft and axial sensors, and the value ranges are [1,p-2] and [1,q-2]. Formula (9) can be obtained by substituting Formula (5) into (8).

(9) $$\left\{ \begin{array}{l} \varpi (C_{i})=X_{c_{i}}=X-F_{W}\;in\;which,F_{W}=\frac{D_{C}}{\sqrt{\frac{P_{w_{i+1}}}{P_{w_{i}}}}+1},i\in [1,p-2],X\in [\frac{w}{2},W-\frac{w}{2}],F_{W}\in (0.489,0.978)\\ \alpha (R_{j})=Y_{R_{j}}=Y-F_{Z}\;in\;which,F_{Z}=\frac{D_{R}}{\sqrt{\frac{P_{z_{j+1}}}{P_{z_{j}}}}+1},j\in [1,q-2],Y\in [l,L-l],F_{Z}\in (0.162,0.328) \end{array}\right.$$

In Formula (9), $X_{c_{i}}$ and $X_{Rj}$ represent the x-axis coordinates of the *i*th weft CNT yarn sensor and the y-axis coordinates of the *j*th axial sensor, respectively; $P_{w_{i}}$ and $P_{w_{i+1}}$ represent stress concentration values measured by the *i*th and *i*+1th weft sensors, respectively; $P_{zj}$ and $P_{zj+1}$ represent stress concentration values measured by the *j*th and *j*+1th weft sensors, respectively.

Through the analysis of the stress sensitivity Formula (2) of the sensor, it is found that when the stress increases from 0 MPa to 100 MPa, the sensitivity range of CNT yarn sensor is [100 MPa, 200 MPa], which is 2/3 higher than the original value. In order to ensure that the sensor works within a high sensitivity range, the force detection range of the stress concentration of the damage source was limited to [100 MPa, 200 MPa ]. Within this range, combined with the mathematical model analysis of the distribution law of stress concentration, it was concluded that when the stress concentration was distributed along the axial and weft directions of the specimen, the effective detection distances $D_{C}$ and $D_{R}$ were between [1.18 cm, 1.67 cm] and [0.39 cm, 0.56 cm], respectively. Through further analysis of Formula (9), it was concluded that the value range of $P_{w_{i}+1}/P_{w_{i}}$ and $P_{z_{j+1}}/P_{z_{j}}$ was between [0.5, 2]. When $D_{C}$ took the minimum value and $P_{w_{i+1}}/P_{w_{i}}$ took the maximum value, the minimum value of $F_{W}$ was 0.489. When $D_{C}$ took the maximum value and $P_{w_{i+1}}/P_{w_{i}}$ took the minimum value, the maximum value of $F_{W}$ was 0.978. When $D_{R}$ took the minimum value and $P_{z_{j+1}}/P_{z_{j}}$ took the maximum value, the minimum value of $F_{Z}$ was 0.162. When $D_{R}$ took the maximum value and $P_{z_{j+1}}/P_{z_{j}}$ took the minimum value, the maximum value of $F_{Z}$ was 0.328.

#### 4.3.3. Coverage Constraint Function of CNT Yarn Sensor Network

CNT yarn sensor is a linear sensor. In the process of locating the damage source, the axial and weft sensors respectively monitored the distribution of stress concentration along the weft and axial directions of the specimen. The monitoring coverage was a rectangular area. Taking the axial CNT yarn sensor as an example, when the detected stress concentration value was set in the range of [100 MPa, 200 MPa], the detectable area interval should be the distance of stress concentration along the material weft distribution multiplied by the length of the specimen, which was [L×0.39cm,L×0.56cm]. Similarly, the detectable area of the weft CNT yarn sensor was [W×1.18cm,W×1.67cm]. Only when a certain number of axial and latitudinal sensors were interlaced horizontally and longitudinally and the detection coverage area was equal to the area of the specimen, the full coverage of the specimen damage location can be realized. However, due to the limitation of CNT yarn sensor embedding process, the edge of the specimen cannot be embedded with the sensor. Therefore, on the condition that the coverage requirements were met, the sensors at the edge shall be embedded at the positions where the embedding was allowed to the greatest extent possible. Set the detection coverage of a single axial and weft CNT yarn sensor as $P(C_{i})$ and $P(R_{j})$, respectively, and the coverage is shown in Formula (10):

(10) $$\left\{ \begin{array}{l} P(C_{1})=P(C_{2})=\cdots P(C_{i})=P(C)=\frac{D_{C}}{L}\;in\;which,D_{C}\in [1.18,1.67],i\in [1,p-2]\\ P(R_{1})=P(R_{2})=\cdots P(R_{j})=P(R)=\frac{D_{R}}{W}\;in\;which,D_{R}\in [0.39,0.56],j\in [1,q-2] \end{array}\right.$$

The joint coverage of the axial and latitudinal sensor networks on the test piece can be obtained from Formula (10), as shown in Formula (11):

(11) $$\left\{ \begin{array}{l} P(C_{1}\cup C_{2}\cup \cdots \cup C_{i})=1-{\left(1-\frac{D_{C}}{L}\right)}^{i}\;in\;which,D_{C}\in [1.18,1.67],i\in [1,p-2]\\ P(R_{1}\cup R_{2}\cup \cdots \cup R_{j})=1-{\left(1-\frac{D_{R}}{W}\right)}^{j}\;in\;which,D_{R}\in [0.39,0.56],j\in [1,q-2] \end{array}\right.$$

When the axial and weft CNT yarn sensors are interlaced horizontally and vertically to form a detection network, the damage source of the specimen can be located. The total coverage P of the CNT yarn sensor network is the product of the axial and weft sensor coverage, as shown in Formula (12). Formula (12) is used as the constraint function of the total coverage of CNT yarn sensor network, which together with Formulas (9)–(11) constituted the constraint function in the NNIA algorithm:

(12) $$P=1-{\left(1-\frac{D_{C}}{L}\right)}^{i}-{\left(1-\frac{D_{R}}{W}\right)}^{j}+{\left(1-\frac{D_{C}}{L}\right)}^{i}{\left(1-\frac{D_{R}}{W}\right)}^{j}$$

### 4.4. NNIA Solution of CNT Yarn Sensor Optimization Problem

The optimized configuration results obtained from the calculation of test pieces with different sizes and specifications using the NNIA sensor network optimization configuration algorithm are shown in Table 4. Through the analysis of the calculation results, it was found that when the length and width of the specimen were less than 10 cm, the coverage rate of the sensor network to the specimen was less than 80%. This was due to the fact that the sensor cannot be embedded at the edge of the specimen in the axial direction. In the weft direction, a maximum of two sensors can be embedded in a knot length. Therefore, in practice, the maximum area in which sensors can be embedded is limited. No matter how many sensors are added in the area, the detection coverage of the sensor network cannot be improved. When the specimen size was greater than 10 cm × 10 cm, the detection coverage rate of the sensor network can basically reach more than 90%. With the increase in the specimen size, the influence of the embedding process of the sensor on the coverage has become very small, and the main factor affecting the coverage has become the stress sensitivity of the sensor. Under the action of low stress, the sensitivity value of the sensor is limited. When the stress is greater than 200 MPa, the sensor can better sense the stress. This was also the reason why the coverage rate of the sensor network of the specimen with a size of more than 10 cm × 10 cm under the low stress state does not reach the maximum coverage rate that the embedding process can meet.

**Figure 7:**
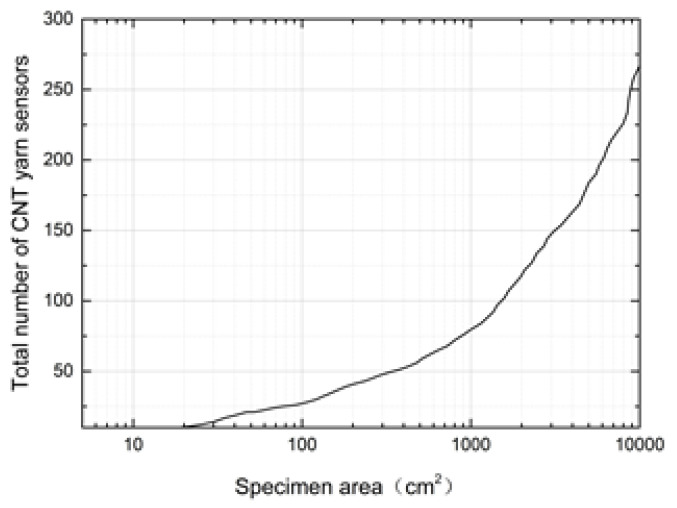
Relationship between the area of the specimen and the total number of optimized CNT yarn sensors.

Through the data analysis of the number of sensors embedded, it was found that when the specimen size was less than 10 cm × 10 cm, there were three combinations of axial and latitudinal sensors, since there was a non-dominant relationship between the coverage and the number of sensors. For example, the size of the test piece was 8 cm × 1 cm, when the number of sensors was {2,6}, the coverage rate was 74.6%; and when the number was {2,5}, the coverage rate was 70.4%. In order to achieve a higher coverage rate, {2,6} should be selected as the optimal solution of the sensor configuration, which was more conducive to the detection and location of the damage source. With the increase in specimen size, the result with the least number of sensors and the highest coverage should be selected as the optimal solution, which was more conducive to weight reduction and cost reduction. The relationship between the area of the specimen and the optimized total number of the corresponding axial and weft CNT yarn sensors is shown in Figure 7.

It can be seen in Figure 7 that the number of CNT yarn sensors increases exponentially with the increase in specimen area. When the specimen area was within the range of 5~40 cm^2^, the total number of sensors embedded was between 7 and 19; when the area of the specimen was within the range of 40~1000 cm^2^, the total number of sensors was between 19 and 79; when the area of the specimen was within the range of 1000–10,000 cm^2^, the total number of sensors was between 79 and 267.

After optimizing the configuration of CNT yarn sensors embedded in braided composites using NNIA algorithm, the eigenvalues of the specimens were decomposed by the singular value decomposition method of quartered matrix. The damage coordinates of specimens 1 to 4 were calculated by comparison with the characteristic values of specimen 5 without damage. The actual damage coordinates of specimens (specimens 1 to 4) were obtained by ultrasonic C-scan technology. The comparison between the calculated coordinates and the actually measured coordinates is shown in Table 5. The results showed that the maximum deviation between the predicted coordinates and the actual measured coordinates was less than 1 mm. This showed that the CNT yarn sensor embedded in the optimized configuration scheme can basically realize the accurate location of the damage of the three-dimensional braided composites.

## 5. Conclusions

Based on the analysis of the 3D six-direction braiding process, the sensitivity of CNT yarn sensor and the distribution of the stress concentration of the damage source of the three-dimensional braided composite, a multi-objective optimization algorithm of NNIA was constructed. The number and position of CNT yarn sensors embedded in 3D braided composite specimens with different sizes were solved. Compared with the actual damage coordinates of the specimen obtained by ultrasonic C-scan technology, it was found that the embedded CNT yarn sensor could accurately locate the damage through the quadrant matrix singular value decomposition algorithm after the optimized configuration of NNIA algorithm, and the maximum positioning error was less than 1 mm. For our research group, Dong Qingxia used a 10-um carbon nanotube yarn sensor to detect the damage of three-dimensional braided composites, and calculated the location error of the damage position by the quadripartite matrix singular value decomposition algorithm to be 5 mm [31]. Compared with the author’s method, the method of locating error in this paper was reduced by 5 times. In conclusion, this method better solves the essential technical problems of sensor embedding, and lays a foundation for the establishment of damage source positioning model of 3D braided composites.

## Figures and Tables

**Table 1:** Proportion and viscosity of resin system.

Name	Epoxy Resin	Curing Agent
Type	JL-155	JH-196
Epoxy value (mol/100 g)	0.779	-
Density (g/cm^3^)	1.13	0.94
Viscosity (MPa·s 25 °C)	618.9	47.2
Weight ratio	75%	25%
Mixed viscosity (MPa·s 25 °C)	271	

**Table 2:** Model of specimen.

Name	Value
Model of braiding yarn	T300-12K
Linear density of braiding yarn (g/m)	0.8
Specimen size (mm)	180 × 40 × 4
Braiding angle (°)	19.8 ± 3
Knot length (mm)	10
Knot width (mm)	2

**Table 3:** The specification parameters of CNT.

Name	Value
Diameter (μm)	100
Density (g/cm^3^*)*	0.8
Elongation at break	20–30%
Strength (MPa)	310–500
Modulus (GPa)	10

**Table 4:** Optimal configuration of CNT yarn sensors embedded in different size specimens.

Specimen Size (cm)	Total Coverage of Sensor Network	Number of Axial Sensors	Number of Weft Sensors	Axial Sensor Position	Weft Sensor Position
6 × 1	69.5%	2	4	{1,7}	{1,5,8,11}
6 × 1	62.9%	2	5	{1,7}	{1,3,7,10,12}
6 × 1	70.1%	3	4	{1,4,7}	{1,3,7,10,12}
6 × 2	77.8%	4	4	{2,8,14,19}	{1,5,8,11}
6 × 2	80.4%	4	5	{2,8,14,19}	
6 × 2	79.5%	5	4	{1,5,10,15,19}	{1,5,8,11}
8 × 1	74.6%	2	6	{1,7}	{1,4,7,10,13,16}
8 × 1	70.4%	2	5	{1,7}	{1,5,8,13,16}
8 × 1	71.2%	3	5	{1,4,7}	{1,5,8,13,16}
10 × 10	90.4%	20	7	{1,3,5,7,9, 11,13,15,17,19}	{1,4,7,10,13,16,19}
20 × 20	91.8%	40	13	{1,6,11,16,21,26,31,36,41,46,51,56,61,66,71,76,81,86,91,96,100,105,110,115,120,125,130,135,140,145,150,155,160,165,170,175,180,185,190,195,200}	{1,4,7,11,14,17,21,24,27,31,34,37,40}

**Table 5:** Predicted and actual coordinates of damage in braided composites.

Name of Specimen	Name of Damage Source	Predicted Damage Coordinates (x,y)	Actual Damage Coordinates $\left(x^{\prime },y^{\prime }\right)$	Maximum Deviation between Predicted and Actual Damage Coordinates (mm) $\sqrt{{\left(x-x^{\prime }\right)}^{2}+{\left(y-y^{\prime }\right)}^{2}}$
1	1-1	(9.1,84.2)	(8.8,84.9)	0.76
1	1-2	(13.4,97.8)	(12.6,97.3)	0.94
2	2-1	(9.1,88.5)	(9.6,88.9)	0.64
2	2-2	(15.3,104.7)	(15.5,105.1)	0.45
3	3-1	(15.3,94.6)	(16.0,94.1)	0.86
4	4-1	(12.5,92.6)	(12.1,93.0)	0.57
